# Multi-omics analysis of NET+ TAN explains the immunosuppressive TME and prognosis value of malignant clinical characteristics in TNBC

**DOI:** 10.1016/j.tranon.2026.102692

**Published:** 2026-02-10

**Authors:** Qiannan Zhu, Xiangxin Zheng, Xiaochao Zhu, Peng Yang, Mengzhu Yang

**Affiliations:** a Department of Breast Surgery, The First Affiliated Hospital with Nanjing Medical University, Nanjing, Jiangsu Province, 210000, China; b Department of Breast and Thyriod Surgery, Suqian First Hospital, Suqian, Jiangsu 223800 China; c Department of Oncology, The First Affiliated Hospital with Nanjing Medical University, Nanjing 210029, Jiangsu, China

**Keywords:** TNBC, NETs, TME, Prognosis value, Multi-omics analysis

## Abstract

Triple-negative breast cancer (TNBC) continues to exhibit a poor response to both immunotherapy and endocrine therapy, primarily due to its complex genetic heterogeneity and tumor immune microenvironment (TIME). Neutrophil extracellular traps (NETs) are involved in neutrophil development and degranulation in treatment-resistant tumors, particularly TNBC and metastatic cases. Using a combination of single-cell RNA sequencing (scRNA-seq) datasets, bulk mRNA sequencing data from The Cancer Genome Atlas (TCGA) cohort, microarray data from the METABRIC and GEO databases, and epigenetic sequencing resources, we comprehensively investigated the functional characteristics and prognostic potential of NET-positive tumor-associated neutrophils (NET+ TAN) in TNBC. A six-gene panel derived from NET+ TAN has been identified as a reliable diagnostic biomarker for the survival of patients with TNBC and has been validated across several independent cohorts. Notably, our findings indicate that NET+ TAN facilitate T cell reprogramming in TNBC, thereby establishing a suppressive TIME and promoting tumor progression. This study provides a comprehensive perspective on the significance of NETs in patients with TNBC, with the aim of offering genetic and epigenetic insights into the mechanisms by which NETs may transition from cold to hot tumor phenotype, as well as valuable recommendations for therapeutic strategies.

## Highlights

• NET+ TAN identified as driver of immunosuppressive tumor microenvironment in TNBC, linked to poor prognosis and tumor progression.
• A novel six-gene signature derived from NET+ TAN shows robust prognostic value across multiple cohorts, with high clinical utility for TNBC survival prediction.
• Super-enhancer analysis reveals key transcriptional regulatory networks underlying NET+ TAN activity, highlighting SLC24A4 as a promising therapeutic target.
• Single-cell analysis demonstrates NET+ TAN repolarizes T-cells toward immune-suppressive phenotypes, elucidating mechanisms of immunotherapy resistance.
• Integrative multi-omics approach uncovers novel epigenetic and transcriptional regulatory mechanisms governing TNBC immune microenvironment remodeling.

## Graphic abstract

**Figure:**
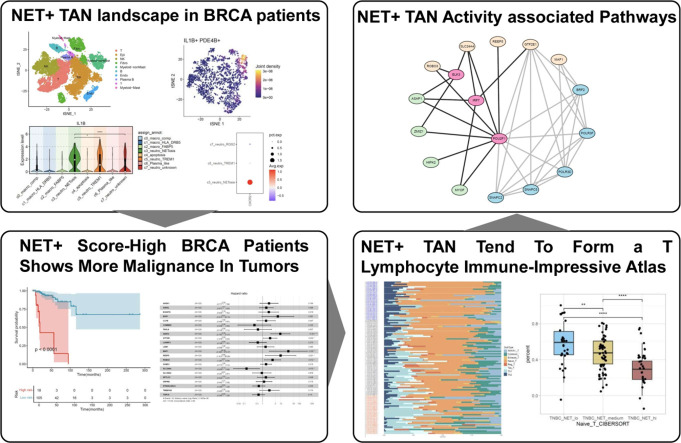

## Introduction

Breast cancer (BRCA) is the most frequently diagnosed malignancy and leading cause of cancer-related mortality among women globally[1]. Among the various molecular subtypes of BRCA, triple-negative breast cancer (TNBC), characterized by a lack of the expressions of the estrogen receptor (ER), progesterone receptor (PR), and human epidermal growth factor receptor 2 (HER2)—constitutes a particularly aggressive category, accounting for 15–20 % of all cases[2,3]. TNBC is associated with distinct clinical features including elevated rates of distant metastasis and a significantly poorer prognosis [[4], [5], [6]]. Therefore, identifying potential biomarkers for the diagnosis and treatment of TNBC is urgently needed.

Neutrophils are the most abundant myeloid-derived cells in the peripheral circulatory system and have garnered significant attention in cancer research. Tumor-associated neutrophils (TANs) exhibit high plasticity and are traditionally classified into two functional groups: N1, which possesses anti-tumor properties, and N2, which exhibits pro-tumor characteristics. For example, exosomes secreted by metastatic bone tumors contain *DKK*1, which hinder the maturation of TANs and reduces their sensitivity to immune checkpoint blockade therapy. Numerous studies have demonstrated that *PD-1* immune therapy can significantly increase the proportion of N2-type TANs in malignant tumors, including metastatic tumors. This increase interacts with *PD-1* expressing T cell subtypes, primarily exhausted T cells, resulting in diminished T cell chemotaxis. Furthermore, TANs have been reported to express and secrete immunosuppressive molecules such as *ARG1* and *CCL17*[7], which inhibit cytotoxic T cell proliferation, and *VEGF* and *MMP9*[8], which contribute to adverse angiogenic tumor physiology. Owing to a lack of effective clinical immunotherapy or chemotherapy options and its ability to evade the immune system, TNBC has attracted considerable attention. 5-HT induces histone serotonylation (H3Q5ser) and orchestrates histone citrullination (H3cit) in neutrophils, triggering chromatin decondensation, and facilitating the formation of neutrophil extracellular traps (NETs) [9]. Histone methyltransferase *EZH2* and histone *H3K36* trimethyltransferase *SETD2* are vital epigenetic factors that regulate neutrophil infiltration during cancer metastasis [10,11]. These findings suggest that neutrophils respond to different epigenetic signals [12].

Recently, super-enhancers (SEs) have gained attention because of their pivotal roles in regulating gene expression and driving abnormal transcriptional programs in tumors [13]. These composite elements, which consist of numerous enhancers and transcription factors, exhibit high H3K27ac levels, strong transcription factor binding, and significant regulatory effects on target genes [[14], [15], [16]]. SEs are crucial for tumorigenesis, drug resistance, metastasis, and tumor microenvironment[[17], [18], [19], [20]]. In breast cancer, PDL1L2-SE has been linked to PD-L1 and PD-L2 overexpression and immune evasion[21]. SEs are also associated with invasion, metastasis, and the TNBC microenvironment, specifically regulating genes such as FOXC1, MET, and ANLN, contributing to oncogene dysregulation[22]. Increasing evidence indicates that SEs play a pivotal role in the development of various cancers. However, the functional characteristics and clinical significance of SEs in TNBC have not been thoroughly explored.

NETs are triggered by changes in the tumor microenvironment and consist of proteins, such as *ELANE, IL1β, APRIL*, and DNA-histone complexes. They promote cancer cell growth and inhibit cytotoxic lymphocyte growth. Recent studies highlight the role of NET+ tumor-associated neutrophils (TAN) in triple-negative breast cancer (TNBC) progression and metastasis. Arachidonic acid disrupts lipid metabolism in TAN leading to resistance to PD-1 therapy in TNBC. NET+ TAN are more prevalent in TNBC than in other types of breast cancer. Similarly, in a TNBC lung metastasis study, NETs were more common in triple-negative tumors than in luminal tumors. In a murine 4T1 breast cancer model, Teijeira et al. found that tumor-derived *CXCR1* and *CXCR2* agonists induced the production of NETs, which encapsulated tumor cells and prevented them from contacting immune cells, thereby inhibiting the tumoricidal effects of cytotoxic T lymphocytes (CTLs) and NK cells and occupying an immunosuppressive niche [23]. *CHI3L1*, a secretory molecule from cancer epithelial cells, especially TNBC, has emerged as a key factor for reshaping TAN into NET+cells, thus preventing the immune response to PD-1 immune therapy[24,25]. Although TAN is an established biomarker in breast cancer and TNBC, the comprehensive observation of how TAN related TIME/TME influences the survival of TNBC, the prognostic value of TAN in TNBC patients, and the underlying mechanism of transcriptional and epigenetic regulation of intrinsic TAN molecular programming remains largely unclear. In this study, we evaluated the prognostic value of TAN in patients with TNBC and the transcriptional and epigenetic regulatory networks underlying TAN.

## Materials and methods

### Pre-processing of single-cell RNA-sequencing data of public pRCA cohort

The downloaded expression matrix was imported into the Seurat (v5.3.0) R toolkit (R version 4.4.0) for quality control and downstream analysis of the singlecell RNAseq data. We first filtered the relatively-loose metrics to exclude low-quality cells and to keep neutrophils as much as possible, following the following relatively-loose criteria: (1) the number of detected transcripts (number of unique UMIs) was more than 0; (2)the number of detected genes was more than 100; and (3) the percentage of reads mapped to mitochondrial genes was no more than 50 %. The percentage of mitochondrial gene expression was calculated using the PercentageFeatureSet function in the Seurat R package. For further analysis, data normalization was performed using default parameters. The FindVariableFeatures function in Seuratpackage was performed for extracting a subset of variable genes (selection.method = 'vst,’ nFeatures = 2000). We then performed principal component analysis(PCA) with the parameters of dims=1:20 after scaling the data with the ScaleData function in Seurat, using all detected genes as input features. Next, we integrated data from different samples using the RunHarmony method in the R package harmony (v1.2.3) with default parameters. The clusters were visualized on a 2D map produced with UMAP using the DimPlot function in Seurat.

### Identification of cell types and subtypes using t-SNE

The cells were clustered by graph-based clustering of PCA reduced data using the RunTSNE function. For sub-clustering, we used the FindClusters function (resolution=1) in Seurat. For each cluster, we used the Wilcoxon Rank-Sum Test to identify significantly differentially expressed genes compared with the remaining clusters (min.pct = .25, logfc.threshold = .25). Canonical markers for possible celltype were used for DotPlot in Seurat using slot 'data'.

### Celltype sub-clustering and exploration

Re-clustering of all Myeloids and T lymphocytes was performed using the same workflow as previously mentioned, with the exception of using the FindClusters function with resolution = 0.5 for myeloid cells and 2 for T cells. A heatmap plot of representative genes was generated using pheatmap (v1.0.12) in R, and an expression dot plot was generated with the DotPlot function in Seurat. To infer the developmental traces of T cell subtypes between the two sample groups, we utilized hvg (dispersionTable function in MONOCLE V2) as the input gene. Cell-cell communication analysis of myeloid and T cells was completed with CellChat (v1.6.1) using default parameters in R. Violin plot illustrating gene expression levels in scRNA-seq was performed with FeatureStatPlot in the R package SCP(v0.5.1). Evaluation of the CancerSEA gene set activity score in scRNA-seq dataset was performed using AUCell(v1.28.0) in R. Cell type proportion deconvolution of TCGA-TNBC patients was performed using the CIBERSORT svr method embedded in the IOBR(0.99.9) package. The SCENIC(v1.3.1) standard workflow in the R package was used with the default parameters.

### Analysis of bulk RNA-Sequencing expression data from TCGA-BRCA cohort

Differential analysis was performed using DESeq2(v1.40.2) in the R software. The selection criteria for differentially expressed genes (DEGs) were as follows: fold change > 1.5 or fold change < 1/1.5, and p < 0.05. Functional analysis and network visualization of all genes mentioned in this paper were performed using clusterProfiler (v4.14.6) and the in-house ggplot2 script in the R package (v3.5.2). Gene set activity calculations in the bulk RNA-seq dataset were implemented using the ssGSEA method in the R package GSVA(v1.48.3).

### Survival, prognosis, and validation analysis of TCGA mRNA expression cohorts

RandomSurvivalForest analysis was implemented in R package randomForestSRC(v3.4.1), Cox regression was performed in R package survival(v3.5.8), and surv_cutpoint and Kaplan-Meier(K-M) survival plots were completed with ggsurvplot function in R package survminer(v0.5.0).

### ChIP-seq data analysis, superenhancer and transcription factor identification

Raw fastq sequence files from the GEO datasets GSE262487 for the MDA-MB-231 cell line and GSE260793 for the T47D cell line were downloaded, and adaptor trimming was executed with trim-galore(v2.8) with parameters -j 4 -e 0.2 –length 20 –stringency 3 –paired. Quality-controlled clean reads were mapped to the human hg38 database using the default parameters of bowtie2(v2.5.2). Subsequently, SAM files were processed with samtools sort and index function. After the necessary preliminary analysis procedure, MACS3(v3.0.3) callpeak was implemented to identify potential enhancers. Then,the Python script ROSE was utilized to obtain the stitched super-enhancers (SEs). The SE peak annotation was completed using the peakAnno function in the R package ChIPseeker(v1.42.1). SE peak signal curve was plotted with computeMatrix+plotHeatmap in deepTools(v3.5.6).

Up-stream TF prediction of target genes was implemented in R package cisTarget(v1.20.0), coupled with motif database 'hg38_10kbp_up_10kbp_down_full_tx_v10_clust.genes_vs_motifs.rankings.feather' (https://resources.aertslab.org/cistarget/).

## RESULTS

1. **scRNA-seq analysis unveils the existence of NET^+^ TAN in BRCA patients**
  TAN has been discovered and characterized as an important TME component in many malignant cancers, including cervical cancer[26], colon cancer[27], and non- small cell lung cancer[28]. NET is a canonical phenotype of neutrophils that secrete many immune-regulatory factors such as *IL1β, PAD4* and *ELANE* especially in patients with Breast Cancer (BRCA) with worse clinical outcomes. However, given that the former versions of scRNA-seq platforms, such as 10x genomics, have unavoidable flaws in capturing fragile granulocytes with a rather short half-life and the inherent minor percentage of granulocytes in total cells of the TME, there is still a lack of adequate single-cell RNA sequencing datasets to investigate the TAN subtype in BRCAs.
  Here, we sought to re-analyze one dataset from a pan-cancer cancer-associated fibroblast (CAF) study to dissect TAN subtypes in BRCA [29]. After a less strict quality control procedure in Seurat, we get 35749 cells from 17 patients and found 24 subclusters with a resolution of 1 (Fig. 1A). Subsequently, major cell types, including immune, mesenchymal, and epithelial cells, were identified based on conserved gene signatures(Fig. 1B-D).

**Fig. 1:**
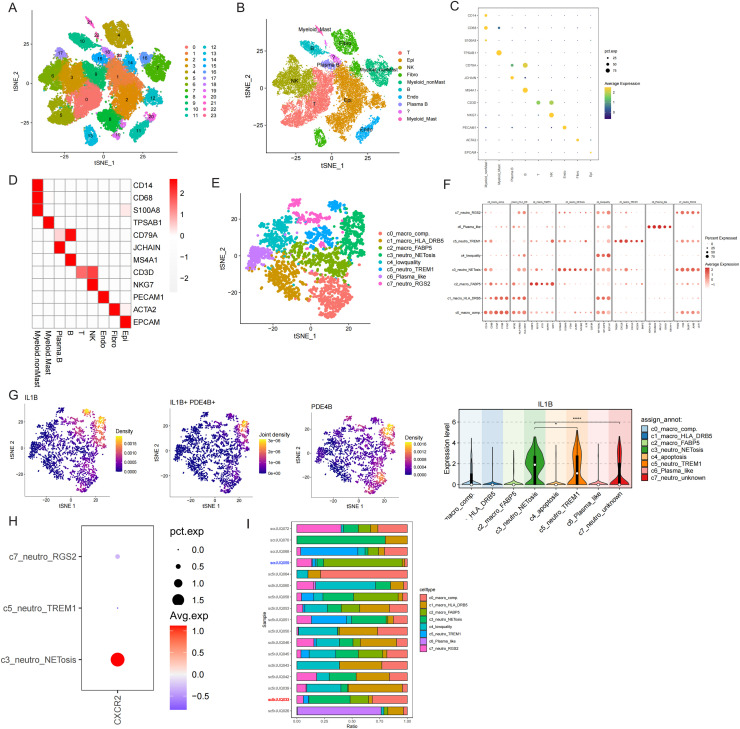
Annotation of scRNA-seq Dataset E-MTAB-8107 And Myeloid Cell Sub-clustering. t-SNE plot of the scRNA-seq dataset showing 24 clusters, and (B) the corresponding annotation result. Dopplot (C) and heatmap(D) of the conserved markers confirm the cell-type resolved annotation.(E) Reclustering and annotation of myeloid cells without mast.(F) Customized markers for each myeloid cluster.(G) *PDE4B* and *IL1β* are two typical markers for NET+TAN.(H) *CXCR2* expression is illustrated as a pro-tumor signature with a dot plot.(I) The bar plot shows the percentage distribution in all patients with BRCA.
  To identify the functional cell subclusters related to NETs, we re-clustered myeloid cells, except mast cells, because of their isolated t-SNE distribution (Fig. 1E-H). As Fig.1E shows, myeloid cells in the E-MTAB-8107-BRCA dataset mainly comprised macrophages and neutrophils, as confirmed by the top expression markers (Fig. 1F). Furthermore, a distinct neutrophil subgroup was identified as NET+ TAN, which was clearly described by the expression of *IL1β* and *PDE4B* (Fig. 1G-H). In some studies related to TME-regulatory plasticity in neutrophils, *ICAM1*(CD54) has been well characterized as an N1-specific TAN marker that exert anti-tumor features and *CXCR2*(CD184) is considered an N2-specific TAN marker with pro-tumor function. As expected, CXCR2 was mainly expressed in NET+ TAN compared to the other two TAN subtypes (Fig. 1H), indicating that NET+ TAN in BRCA mainly exhibit pro-tumor features. Based on myeloid subtype annotation, we summarized the cell count distribution between all samples (Fig. 1I and Supplementary Fig. S1B) and found that the two samples had extreme differences in the NET+ TAN proportion, and both had a relatively high total cell number. This two sample was further regarded as typical 'high versus low' NET+ TAN comparison in many potential aspects such as tumor malignancy, and T cell phenotypes.
2. **NET+ score-high BRCA patients shows more malignance in tumors, especially in TNBC**
  NETs have been widely confirmed in BRCAs in many studies, pointing to the metastasis, differentiation characteristics of epithelial cells, and multi-layer chemotaxis roles of lymphocytes. In order to better understand the probable influence of NET+ TAN on TME, whether it is good or harmful to tumor progression, we firstly tried to investigate several well-reviewed characteristics for cancer epithelial cells based on cancerSEA database.
  As described before, we select two samples in E-MTAB-8107-BRCA dataset as one pair of typical comparison, which sample 'sc5rJUQ033′ has relatively-high NET+ TAN proportion, and 'scrJUQ059′ is on the opposite. It was shown that NET+ high patient shows a rather-higher AUCell in 'Differentiation,' 'Metastasis,' 'EET,' 'Proliferation,' and 'Quiescence' with significant difference under the standard wilcox p < 0.05 in epithelium cells of E-MTAB-8107 cohort (Fig. 2A). We sought to find another scRNA-seq dataset, including NET+ TAN cell-type resolved annotation and complete clinical data, such as molecular typing and genetic information, such as MSI status and medication history, but finally failed with no ideal result. By comparison, we calculated the NET score in the TCGA-BRCA cohort, especially in 123 TNBC patients, because of the significantly higher NET score for TNBC patients compared with non-TNBC patients (Supplementary Fig. S1). As is shown in the results, we adopt a quantile grouping method for NET activity score in TNBC patients, and find that eight tumor malignancy metrics were higher between NET-high and NET-low patients, with only one metric 'EET' showing no significant difference (Fig.2B). ssGSEA method was also applied to the HALLMARK geneset, and many well-established tumor features or signaling pathways also show higher score in NET-high patients, such as KRAS, WNT/beta-Catenin and angiogenesis(Supplementary Fig. S2). These findings indicate that NET+ TAN may act as a vicious source of epithelial cells and facilitate poor clinical outcomes in patients with TNBC.

**Fig. 2:**
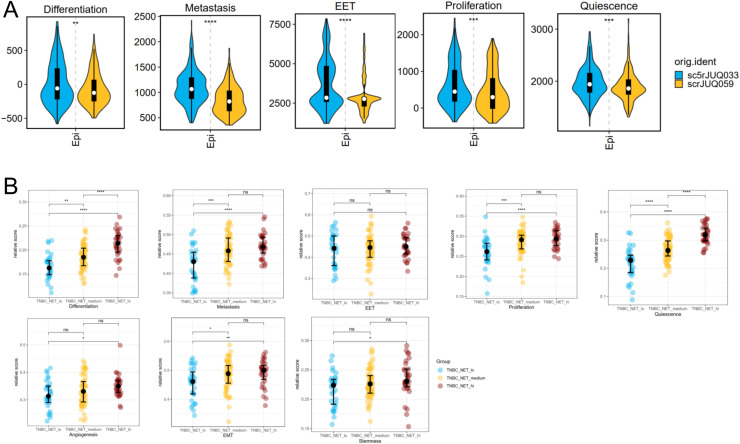
Evaluation of cancerSEA Genesets In BRCA Expression Data.(A) AUCell calculation of cancerSEA geneset score and difference between two representative samples in epithelium of E-MTAB-8107 cohort.(B) ssGSEA score of TCGA-TNBC patients with different level of NET activity. The significance mark '*', '**', '***' and '****' represents wilcox *p* < 0.05, 0.01, 0.001, 0.0001, respectively.
3. **NET+ TAN enriched BRCA patients tend to form a T lymphocyte immune-impressive atlas**
  In addition to the malignant epithelial features, we wanted to determine the extent of NET+ TAN on lymphocytes, such as T, B, Plasma or NK cells. We re-clustered all the T cells in t-SNE plots in E-MTAB-8107 dataset, and got several T subtypes based on the widely-used signatures (Fig.3A-C), including ANXA1+ T(*ANXA1*), Cytotoxic T(*CD8A/CD8B/GNLY*), Exhausted T(*PDCD1/LAG3*), Naive T(*CCR7*), Regulatory T (i.e. Reg T)(*FOXP3*), Tumor Antigen-Specific T Cell (i.e., Tas T) (*CXCL13*), Th1(*IFI44L/IFI6*), Th2(*CXCR4*). Given the insufficient total cell depth and uneven distribution of the scRNA-seq dataset E-MTAB-8107, we aimed to obtain a more comprehensive evaluation of the T cell subtype composition in patients with BC. Based on the svr method in the IOBR R package, we calculated the percentage of the eight T subtypes in TCGA-TNBC cohorts according to the customized gene signature for each T subtype. Among these results, we found that naïve T and Th2, two up-stream sources of cytotoxic T cells, showed a significant decrease in NET-high TNBC in comparison with NET-low individuals, while Tas T and Reg T showed the opposite trend (Fig. 3D-E).

**Fig. 3:**
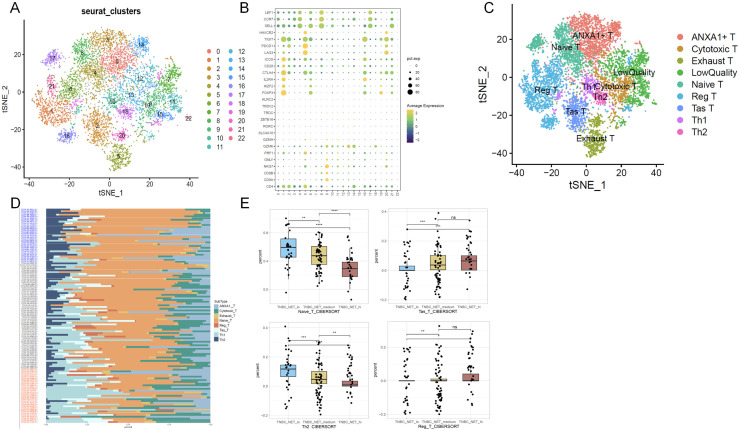
Subtyping of T cells in E-MTAB-8107 Cohort And the Deconvolution Inference of TCGA-TNBC bulkRNA-seq Cohort.(A) t-SNE plot of scRNA-seq dataset divides T cells into 23 natural subtypes and (B) Dotplot of conserved markers leads to cell-type resolved annotation.(C) t-SNE plot shows the functional annotation result.(D) svr method deconvolution in R package IOBR infers the percentage of TCGA-TNBC patients based on the positive markers of NET+ TAN.(E) Statistical analysis of cell proportion in D according to NET activity score. The significance mark '*', '**', '***' and '****' represents wilcox *p* < 0.05, 0.01, 0.001, 0.0001, respectively.
4. **T cell shows different developmental trajectory among NET+ TAN patients**
  For a finer validation of how NET+ TAN prompt chemotaxis and polarization of lymphatic T cells, we performed cell trajectory prediction between T subtypes in the E-MTAB-8107 dataset with MONOCLE2. We found that T cells in BRCA can be divided into two trajectories, taking state4 (mainly composed of cytotoxic and Tas T) as a resource, and one direction is state1 (mainly Exh and Reg T, i.e., immune impressive), while another is state5 (mainly Cytotoxic and Th1/2 T, i.e., immune active) (Fig.4A-B). Then we found that the NET+ TAN high patient 'sc5rJUQ033′ tends to show more Reg T/Exh T developmental potency (Fig. 4C). Extra illustration of several marker genes, to be detailed, FOXP3 for Reg T and LAG3/PDCD1 for Exh T, show higher expression value in 'sc5rJUQ033′ than 'scrJUQ059′. This suggests that myeloid TAN with NET features may facilitate the development of immunosuppressive T cell subtypes (Fig. 4D).

**Fig. 4:**
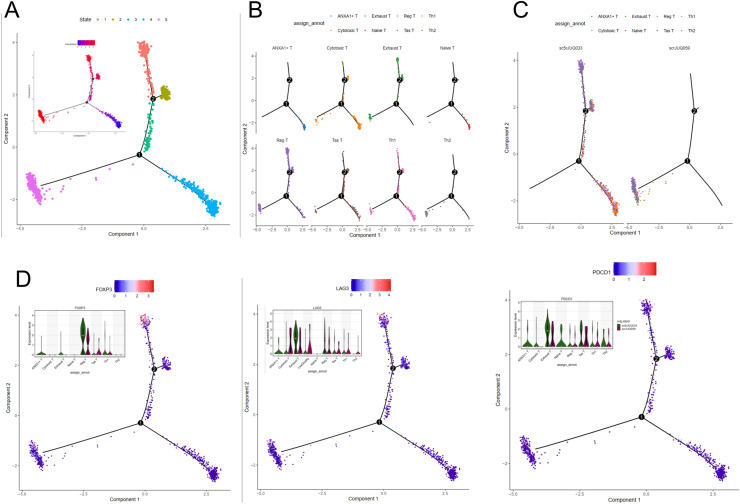
Trajectory Analysis Of T Cell Subtype Among Two Typical Samples. (A) MONOCLE2 analysis shows the pseudotime trajectory and state distribution.(B) and (C) shows celltype- and sample- separated trajectory DD-Tree plot.(D) Expression of signature genes FOXP3 for Reg T and LAG3/PDCD1 for Exhaust T cell are plotted along pseudotime in MONOCLE2.
5. **Cell-Cell Chat inference between TAN and T cell Subtypes explains partial molecular interaction mechanism**
  Based on the observation that NET+ TAN may induce or directly lead to different T cell trajectories, we tried to further investigate the kinds of genes or pathways that participate in this interaction. In this study, we performed cell-cell communication using CellChat in R to explore potential molecular mechanisms. First, we extract the myeloid and T cells from two samples mentioned above and find that 'sc5rJUQ033′ has a higher overall count or strength than 'scrJUQ059′ between myeloid and T lymphocytes (Fig. 5A-B). Specifically, there were more interaction events and differences (ligand-receptor pairs) between source myeloids and target lymphocytes, than between source lymphocytes and target myeloid cells (Fig. 5C-D).

**Fig. 5:**
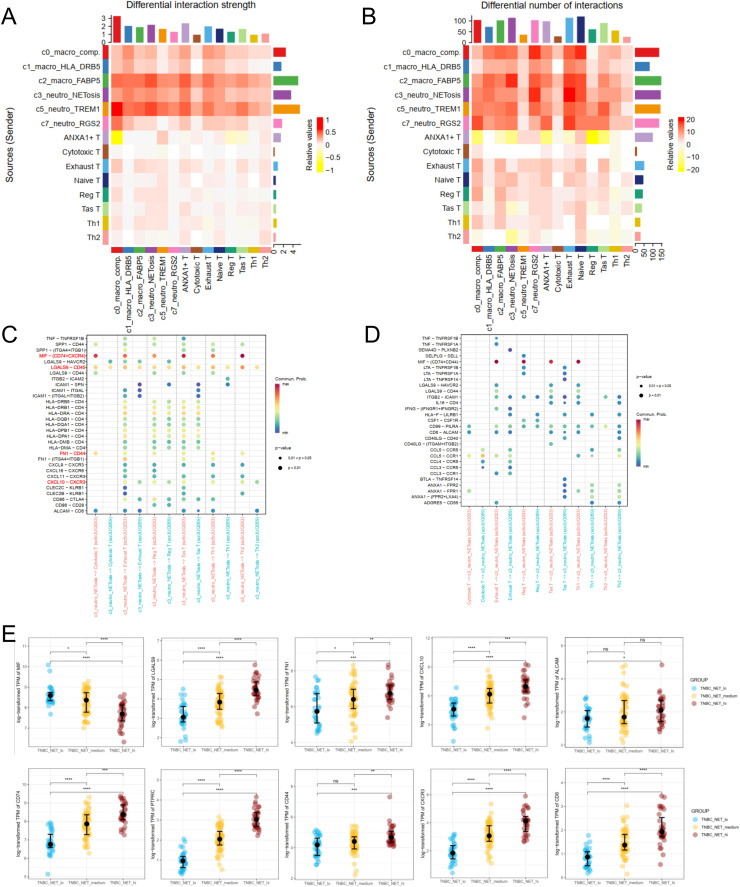
CellChat Analysis of scRNA-seq Dataset And Validation in TCGA-TNBC Cohort. Differential analysis of number(A) and strength(B) in CellChat from sample 'sc5rJUQ033′ -'scrJUQ059′ . (C) Dotplot of communication events taking myeloid cells as the source and lymphocyte T cells as the target, and (D) shows the opposite.(E) Expression of key differentiallly expressed genes between Neutro_NETosis(source) and Reg/Exhaust T(target) in the two samples in (C) was evaluated in TCGA-TNBC patients. The significance mark '*', '**', '***' and '****' represents wilcox *p* < 0.05, 0.01, 0.001, 0.0001, respectively.
  There are many well-known immune-related genes in CellChat, which are highlighted in red in Fig. 5C, including *MIF*-*CD74*/*CXCR4, FN1*-*CD44, CXCL10*-*CXCR3, LGALS9*-*CD45* (*PTPRC*), *ALCAM*-*CD6*. Both *CXCL10* and *CXCR3* play crucial roles in neuroinflammation and neuropathology. CXCR3 is highly expressed in immune and pro-inflammatory cells, whereas *CXCL10* can bind to the cell surface receptor *CXCR3* and conduct immune signaling within the central nervous system. The *CXCL10*-*CXCR3* axis may serve as a therapeutic target for mitigating detrimental events in TNBC cell metastasis to the brain [30]. We also examined the expression data of these genes in TCGA-TNBC cohort and found that all genes were significantly different (Wilcoxon p < 0.05) between NET high and low TNBC patients. It is reasonable to predict that these genes are potentially important in the interaction between NET+ TAN and Reg T/Exhaust T cells (Fig. 5E).
6. **Differential and Functional analysis according to NET+ TAN activity In TNBC reveals the underlying biological pathways**
  Patients were distinctly separated into three groups according to the NET+ TAN activity ssGSEA score in the PCA (Fig. 6A). Differential analysis showed that *IL1β/CXCR*2 for NET+ TAN, *LAG3* for immunosuppressive Reg T, *LAG3* for exhausted T showed significantly higher expression in NET high versus low patients with DESeq2 threshold p < 0.05, FoldChange > 1.5, or FoldChange < 1/1.5 (Fig. 6B). Enrichment analysis against the KEGG and REACTOME databases for upregulated genes also showed that the top-enriched pathways or biological processes were all related to PD1 expression, T cell polarization, cytokine network, and neutrophil phenotypes (Fig. 6C-F), which is in accordance with the CellChat results.

**Fig. 6:**
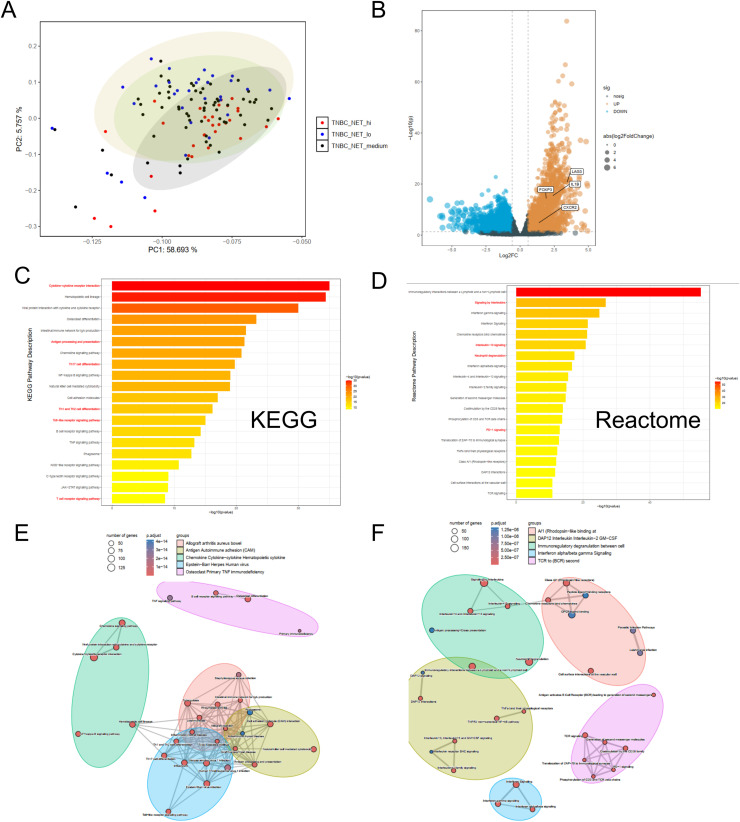
Differential Statistic And Functional Analysis of TCGA-TNBC Patients According To The NET Activity Levels.(A)Unsupervised Principle Component Analysis shows the sample distribution among three NET marker activity.(B) Volcano plot shows the DESeq2 analysis result of NET score high vs low TNBC individuals in TCGA(FoldChange > 1.5 or FoldChange < 1/1.5 and p < 0.05). Highlighted gene text represent markers for NET+ TAN, Exhaust T and Reg T.(C) KEGG and (D) REACTOME enrichment analysis for up-regulated genes between NET-high and low TNBC group.(E) and (F) further exhibits the pathway network of KEGG and REACTOME respectively through the emapplot function in clusterProfiler, which is based on the jaccard similarity.
7. **Identification and validation of clinical value in NET+ TAN among TNBC patients**
  Six genes in NET+ TAN which are significantly related to overall survival(OS) outcome were selected after performing the RSF (Random Survival Forest) + univariate Cox + mult-variate Cox regression workflow among TNBC patients in TCGA-BRCA cohort (Fig. 7B). High expression of GNAT2, GTF2E1, MAF1, REEP5, and ROBO3 acted as risk factors, whereas high expression of SLC24A4 acted as a protective factor against OS (Fig. 7C). We calculated a risk score against six genes according to the expression levels and regression coefficients, and 123 TNBC patients in the TCGA database were stratified into high-and low-risk groups using the surv_cutpoint function in the R package survminer (Fig. 7D). Kaplan-Meier survival curves demonstrated a better survival rate in the low-risk group than in the high-risk group (Fig. 7D). The decision curve analysis (DCA) for survival prognosis is shown in Fig. 7E. ROC analysis was performed using the R package pROC shown in Fig. 7F. Our six-gene integrated model had an AUC above 0.805, which was extraordinarily higher than the ROC value of each individual gene.

**Fig. 7:**
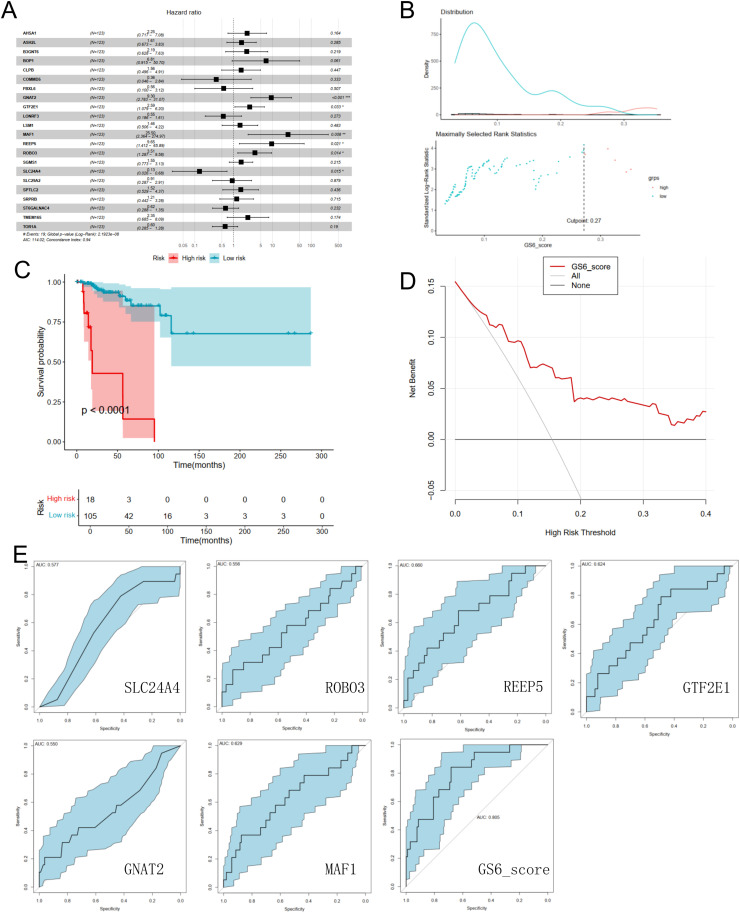
Construction Of The Predictive Risk Model. (A) Forest plot of prognosis-related DEGs, 6 prognostic genes were finally picked after LASSO regression; (B) Distribution of the risk score for the TNBC patients in TCGA database; (C) K-M overall survival curve of prognostic model distribution; (D) Decision curve analysis of prognostic model for survival prognosis; (E) ROC curves in the training set (TCGA-TNBC database).
  Next, we verified the prognostic value of the six genes in the GEO, GSE65194, and METABRIC cohorts to examine the effectiveness of the signature. The K-M survival curves for OS, metastasis time and local recurrence time indicated a better survival probability in the low-risk group than in the high-risk group in the validation set (GSE65194) (Fig. 8A-C, Supplementary Fig. S3A-C). Consistent with the GEO cohort results, survival analyses of the METABRIC cohort also showed that a lower risk score corresponded to a better OS outcome (Fig. 8D, Supplementary Fig. S3D). The area under the ROC curve (AUC) of our model was 0.781 in the GEO cohort and 0.805 in the METABRIC cohort (Fig. 8E-F), implying remarkable effectiveness for predicting clinical features in patients with TNBC, which also has a better prognosis than every single gene in the corresponding cohort.

**Fig. 8:**
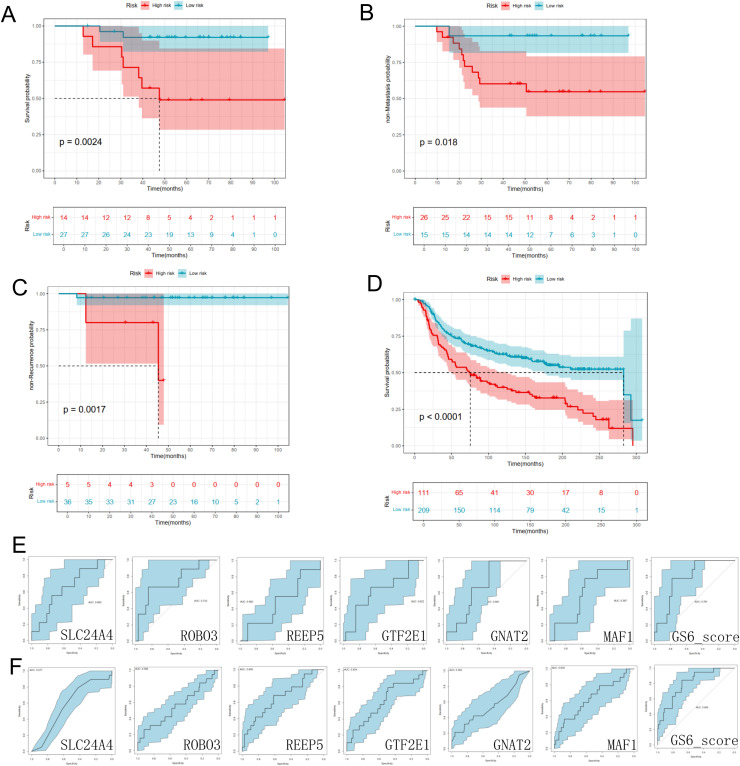
Validation Of Effectiveness In Prognostic Model. K-M survival curves in two validation datasets. Kaplan-Meier analysis of (A) Overall survival (OS), (B) Metastasis time, and (C) Local recurrence time in GSE65194, and (D) OS curves in another validation set (METABRIC) was performed, and the best cut-off values were determined by surv_cutpoint to show the prognostic capacity of the 6 Geneset; The predictive performance of this model was then evaluated for each individual gene and integrated logistic-regression score in GSE65194 cohort (E) and METABRIC cohort (F) by ROC curves.
  In addition to the influence of the six-gene panel on clinical data, we also observed seven typical immune checkpoints and found that all of them showed relatively lower expression in the high-risk group (Fig. 9), which implies that as survival risk increases, the immune characteristics are overall suppressive.

**Fig. 9:**
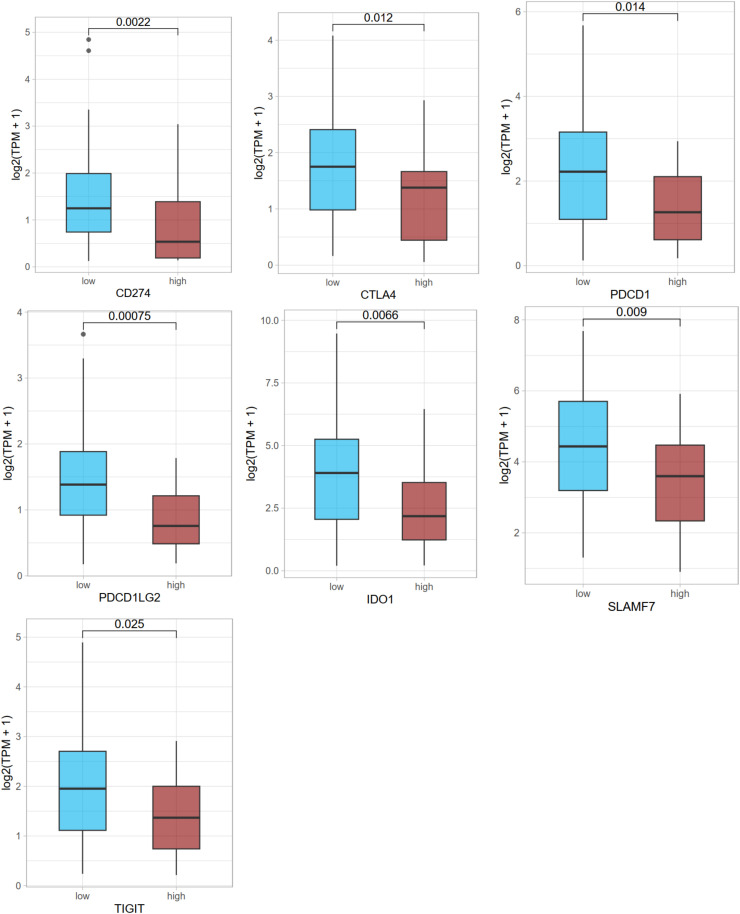
Differences In The Expression Of Immune Checkpoint Genes Between The Survival High- Risk And Low-Risk Groups. The significance mark '*', '**', '***' and '****' represents wilcox *p* < 0.05, 0.01, 0.001, 0.0001, respectively.
8. **Integrative analysis of SEs, transcription factors and survival risk factors re-constructs the prognosis-related gene expression regulatory network**

The six gene score has been validated as a solid survival prognosis criterion, however, what means they are regulated, or through what kind of signaling pathways they act as risk factors remains unclear. We attempted to obtain a comprehensive perspective on the regulatory mechanisms of these important clinical signatures, including TF, SE and possible protein-protein-interaction patterns.

**Fig. 10:**
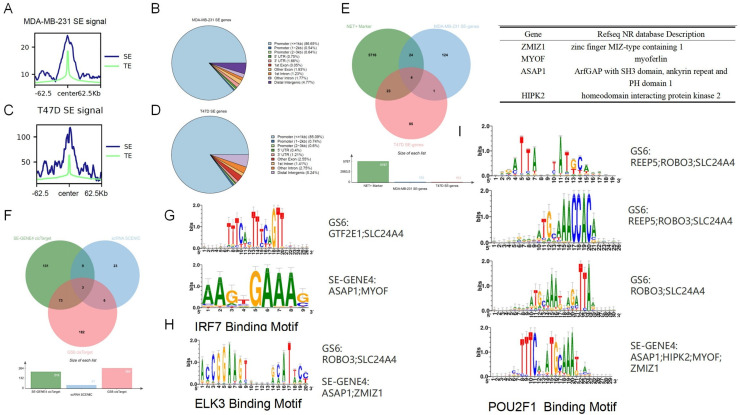
SE And TF Identification Of NET+ TAN Related Risk Genes. Peak curve of SE versus TE in MDA-MB-231(A) and T47D(C) BRCA cell lines. (B) and (D) summarize the percentage of different SE-related genomic regions, which emphasizes promotor as the most enriched and distal intragenic as the second. (E) Venn diagram and functional description for NET+ TAN positive markers, SE-genes in MDA-MB-231 and T47D cell line. (F) Venn diagram of SE-genes-binding TFs, scRNA-seq SCENIC inferred TFs and six-risk-genes-binding TFs. (G-I) illustration of TFs and its binding motif with three target genes.

Recently, SE dysregulation has been demonstrated as an important genetic feature in many types of cancer epithelial cells, such as HNSCC, NSCLC, Neuroblastoma. However, there is still no adequate SE analysis for BRCA or TNBC cell lines. In view of the fact that H3K27Ac is the most investigated epigenetic SE resource, we collected ChIP-seq datasets for two BRCA epithelial cell lines, MDA-MB-231 and T47D. The ROSE algorithm (with a default parameter of 12.5 kb proximity of gene locus from TSS to TTS) was used to call H3K27Ac-enriched SE after peak calling (with MACS3 call peak), in which the promoter region (3 kb up-stream of TSS) was excluded to distinguish Typical Enhancers (TEs) with SE. Finally we get 1865 stitched peaks in MDA-MB-231and 1489 in T47D cells (Supplementary Fig. S4). It was obvious that SE had higher H3K27Ac enrichment than TE (Fig. 10A, C). After mapping SE locus with its related genes using peakAnno function in R package MACS3, it was revealed that 'distal intergenic' was the most abundant SE type, with the exception of Promotor-SEs (Fig. 10B, D).

Subsequently, we combined the SE-genes with NET+ TAN positive markers to explore the extent to which SEs are correlated, and obtained four genes, *ZMIZ1, MYOF, ASAP1* and *HIPK2* (Fig. 10E), and then ZMIZ1 is a zinc-finger TF, HIPK2 is a Ser/Thr protein kinase. After that, to identify possible TFs that can cis-regulate these four genes and/or six survival risk factors, we performed a multi-layer TF prediction procedure:(1) SCENIC prediction of the scRNA-seq dataset including only NET+ TAN (Supplementary Fig. S5), and (2) cisTarget in the R package to predict the potential TF and its binding motif with the corresponding target genes (Fig. 10F). As a result, *IRF7* can bind to the promoter or SE region of *GTF2E1, SLC24A4, ASAP1* and *MYOF* (Fig. 10G); *ELK3* can bind *in-trans* with *ROBO3, SLC24A4, ASAP1* and *ZMIZ1* (Fig. 10H); *POU2F1* can bind to three of six risk genes, *REEP5, ROBO3* and *SLC24A4* through three different conserved DNA motifs, while binding to SE-genes *ASAP1, HIPK2, MYOF, ZMIZ1* (Fig. 10I). These conclusions may highlight a relatively high- level regulatory network, in which NET+ TAN in TNBC intrinsically expresses many TFs and then binds to the DNA motif of target genes, either in the promoter or intergenic region, as well as a fundamentally important gene *SLC24A4*, which was further verified as a reliable candidate for OS in METABRIC TNBC patients using a quantile grouping method (Supplementary Fig. S5). This is consistent with the RSF analysis, which showed higher expression of *SLC24A4* corresponds to better survival.

**Fig. 11:**
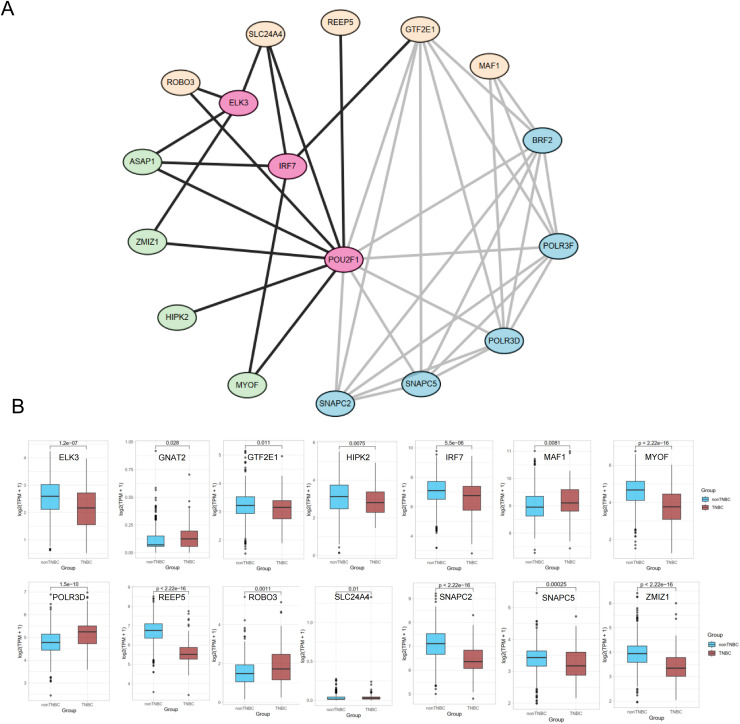
Interated Regulation Network Illustrating the TF-SEgenes-RiskFactor Relationship. (A) The network Plotted In Cytoscape shows a multi-layer gene/protein regulation pattern. (B) Boxplot showing the differences in genes in A between TNBC and non-TNBC patients in TCGA BRCA cohort.

We also extended the PPI network of TFs and target genes using the STRING database and found that *POU2F1* may be a key regulator of survival risk factors and link many important genes (Fig. 11A). It has been reported that *POU2F1* could enhance the proliferation, aerobic glycolysis and the pentose phosphate pathway activity, while reducing oxidative stress and apoptosis in colon cancer cells[31]. In addition, *POU2F1* can promote the metastasis and invasion phenotype of gastric cancer by suppressing the expression of miR-29b1/a cluster[32]. Extra expression comparison of TPM values between TNBC and non-TNBC in TCGA cohort showed different expression patterns of these genes (Wilcoxn p < 0.05) (Fig. 11B).

## Discussion

TNBC poses significant treatment challenges due to its complex composition and relatively poor prognosis[33]. With the elevated appreciation of TIME, there has been an era of novel targeted agents, such as immune check-point inhibitors[3]. However, owing to microenvironment heterogeneity among patients, difficult challenges such as therapeutic resistance, relapse, prognosis and the underlying mechanisms still need to be addressed [34]. Recent studies have shown that TANs are important players in TNBC immunobiology and have shown controversial roles in promoting anti-tumor behaviors through polarization[35]. In our study, using the scRNA-seq dataset, we first obtained intrinsically positive markers for NET+ TAN from myeloids. Compared to the gene signatures mentioned by Li et al[36], which is based on literature curation and mainly related to granulocyte development and migration, the unique genes for NET+ TAN in this paper highlights more immune-regulatory roles, including lymphocyte activation, innate immune system regulation, and inflammatory response (Supplementary Fig. S6). We developed and validated a panel of NET-related scoring markers in patients with breast cancer and found that patients with high NET+ scores demonstrated poorer outcomes. This panel could be used in further analyses to investigate the contribution of NET+ TAN to TNBC. Further analysis found that high-risk patients had immune-suppressive T cell subtypes, with fewer naive T and Th2 cells and more Reg T/Exh T cells among tumor tissues. Further enrichment analysis showed that PD1 expression and cytokine network pathways were involved in NET+ TAN activity. We developed and validated a panel of NET-related scoring markers in patients with breast cancer and found that patients with high NET+ scores demonstrated poorer outcomes. This panel could be used in analyses to further investigate the contribution of NET+ TAN to TNBC. Further analysis found that high-risk patients had immune-suppressive T cell subtypes, with fewer naive T and Th2 cells and more Reg T/Exh T cells among tumor tissues. Further enrichment analysis showed that PD1 expression and cytokine network pathways were involved in NET+ TAN activity.

TAN polarization, characterized by the presence of N1 (anti-tumor) and N2 (pro-tumor) tumor-associated neutrophils, has been increasingly recognized as a key functional factor with the potential to influence the prognosis of patients with breast cancer, especially the TNBC subtype [37,38].. In addition to polarization, neutrophils can combat infections by releasing proteases, superoxides, and NET. However, NETs act as accomplices in tumors[39]. NETs formation is an important function of neutrophils that promotes breast cancer metastasis to the liver and lung[40,41]. In our results, the risk score derived from NET associated genes also showed good ability to distinguish high-risk patients, indicating the potential impact of NET on patients' overall survival. In particular, we found that one of the identified marker genes *GTF2E1* acted as risk factor for TNBC. *GTF2E1*, also known as *TFIIE*, is involved in the transcription of RNA polymerase II[42]. The function of *TFIIE* in transcription initiation is underscored by its interaction with other transcription factors and its role in facilitating the recruitment and activity of the *TFIIH* complex, which can serve as a multi-subunit basal transcription factor that also functions in nucleotide excision repair[43]. TNBC cells require high transcription levels to thrive and maintain their malignant phenotype[44]. Recent studies have shown that *GTF2E1* can be repressed as a target in cancer therapy[45]. For example, the antitumor drug BS-181 is a pyrazolo pyrimidine-derived compound that inhibits the phosphorylation of *Cdk7* targets and the enzymatic functions of *TFIIH*[46].

In our study, we also found that patients with poor prognosis and high NET scores were enriched in Reg T and Exh T cells and diminished in naive T and Th cells. The tumor microenvironment in TNBC is a sophisticated ecosystem comprising immune cells, tumor cells, fibroblasts, and extracellular matrix components[47]. Studies using scRNA-seq have revealed T-cell heterogeneity in TNBC and that regulatory T cells play a key role in immunosuppression and tumor cell immune evasion[48]. This may partially explain the predictive effect of the NET+ risk score. Patients with high-risk scores may have a more immunosuppressive environment in the tumor tissue, leading to a suboptimal response to chemotherapy or immune checkpoint therapy and poor prognosis. In addition, the results indicated that lower-risk patients had higher PD1 expression levels and were more likely to receive immunotherapy. Taken together, our NET+ marker-based scoring model can stratify patients with TNBC and predict their prognosis, potentially affecting their immunotherapeutic choices.

In addition, epigenetic modifications and regulation influence the TNBC tumor microenvironment, such as the SE regulation of target genes. SEs are cis-regulatory elements that promote tumorigenesis and malignancy by altering protein-coding gene expression and noncoding regulatory element functions[49,50]. By integrating H3K27ac ChIP-seq data with the ROSE algorithm, we explored the regulatory networks underlying NETs. We found that four NET-related genes, *ZMIZ1, MYOF, ASAP1* and *HIPK2*, were regulated by TNBC-specific SEs. Interestingly, a previous study reported that *ZMIZ1* interacts with *ER* and *E2F2* to regulate estrogen-responsive cell cycle genes in ER-positive breast cancer, correlating with poor patient prognosis[51]. *MYOF,* a member of the ferlin family, enhances tumor progression and invasion via its regulatory role in membrane transport[52]. Another gene, *ASAP1*, has dual roles in cancer progression; it can promote tumor cell migration and invasion in some contexts, yet acts as a tumor suppressor in ER-positive breast cancer by modulating AKT signaling[53]. While *HIPK2* is a kinase with dual roles in cancer, acting as a tumor suppressor in some contexts and as an oncogenic driver in others, depending on the cancer type and cellular environment. It modulates various signaling pathways and is regulated by cancer-associated miRNAs, making it a potential prognostic marker and therapeutic target[54]. These genes can be utilized as therapeutic targets in future cancer treatment studies.

Taken together, these findings provide a novel scoring model to assess NET+ levels in patients with TNBC and screen suitable patients for immunotherapy. The candidate NET+ related genes and pathways discussed earlier require additional experimental validation and may provide insights for future mechanistic studies. Our results suggest a novel architectural blueprint for TNBC based on the hallmarks of aging in NETs. The NET+ TAN risk score model can optimize TNBC patient survival prediction, support early cancer screening, and guide risk-stratified public health resource allocation.

## Funding

This study was supported by the Young Scholars Fostering Fund of the First Affiliated Hospital of Nanjing Medical University (PY202433), the Jiangsu High-level Hospital Pairing Assistance Research Initiative (JDBFSQ202503).

## CRediT authorship contribution statement

**Qiannan Zhu:** Resources, Methodology, Formal analysis. **Xiangxin Zheng:** Resources, Methodology, Formal analysis. **Xiaochao Zhu:** Writing – original draft, Visualization. **Peng Yang:** Writing – review & editing, Supervision, Funding acquisition. **Mengzhu Yang:** Writing – review & editing, Validation, Supervision, Funding acquisition, Conceptualization.

## Declaration of competing interest

The authors declare that they have no known competing financial interests or personal relationships that could have appeared to influence the work reported in this paper.

## Appendix Supplementary materials

Image, image 1 dummy alt text

Image, image 2 dummy alt text

Image, image 3 dummy alt text

Image, image 4 dummy alt text

Image, image 5 dummy alt text

Image, image 6 dummy alt text

Image, application 7
