# Supplementary figures and images for: Multi-omics analysis of NET+ TAN explains the immunosuppressive TME and prognosis value of malignant clinical characteristics in TNBC
Source: Transl Oncol. 2026 Feb 10;66:102692. doi: 10.1016/j.tranon.2026.102692 (PMC12914808; doi:10.1016/j.tranon.2026.102692)

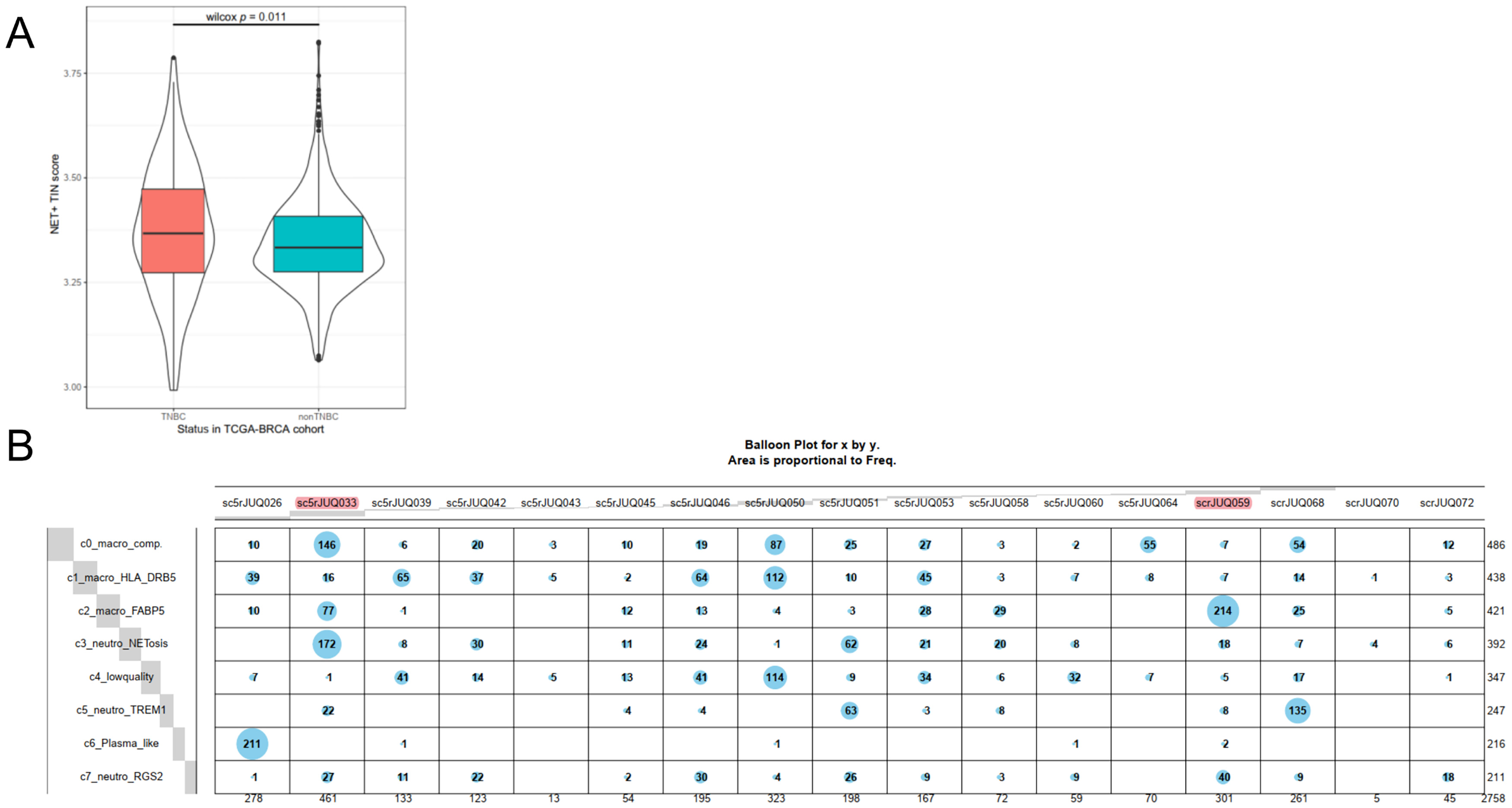

Supplement: Supplementary file 1 [file mmc1.jpg]

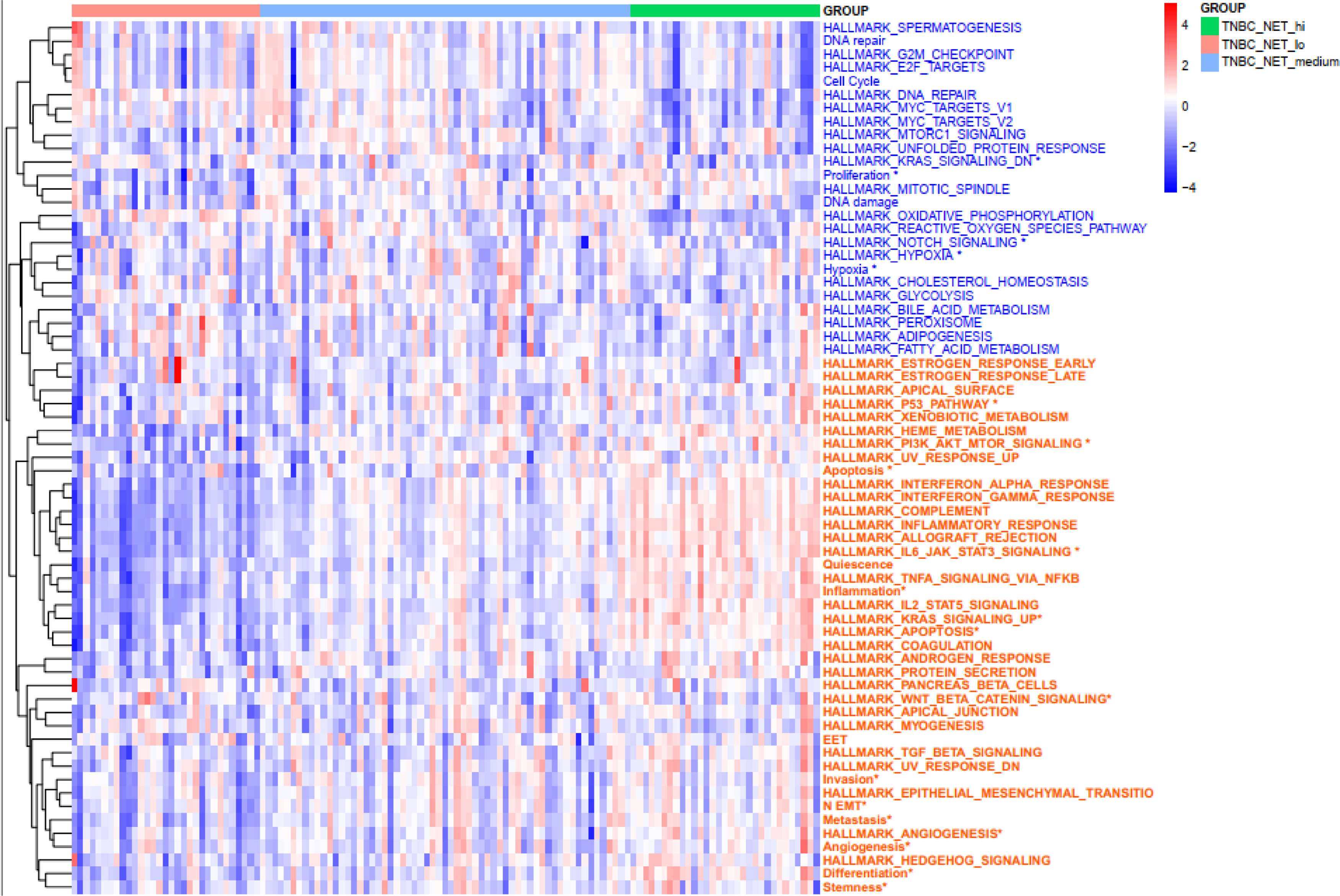

Supplement: Supplementary file 2 [file mmc2.jpg]

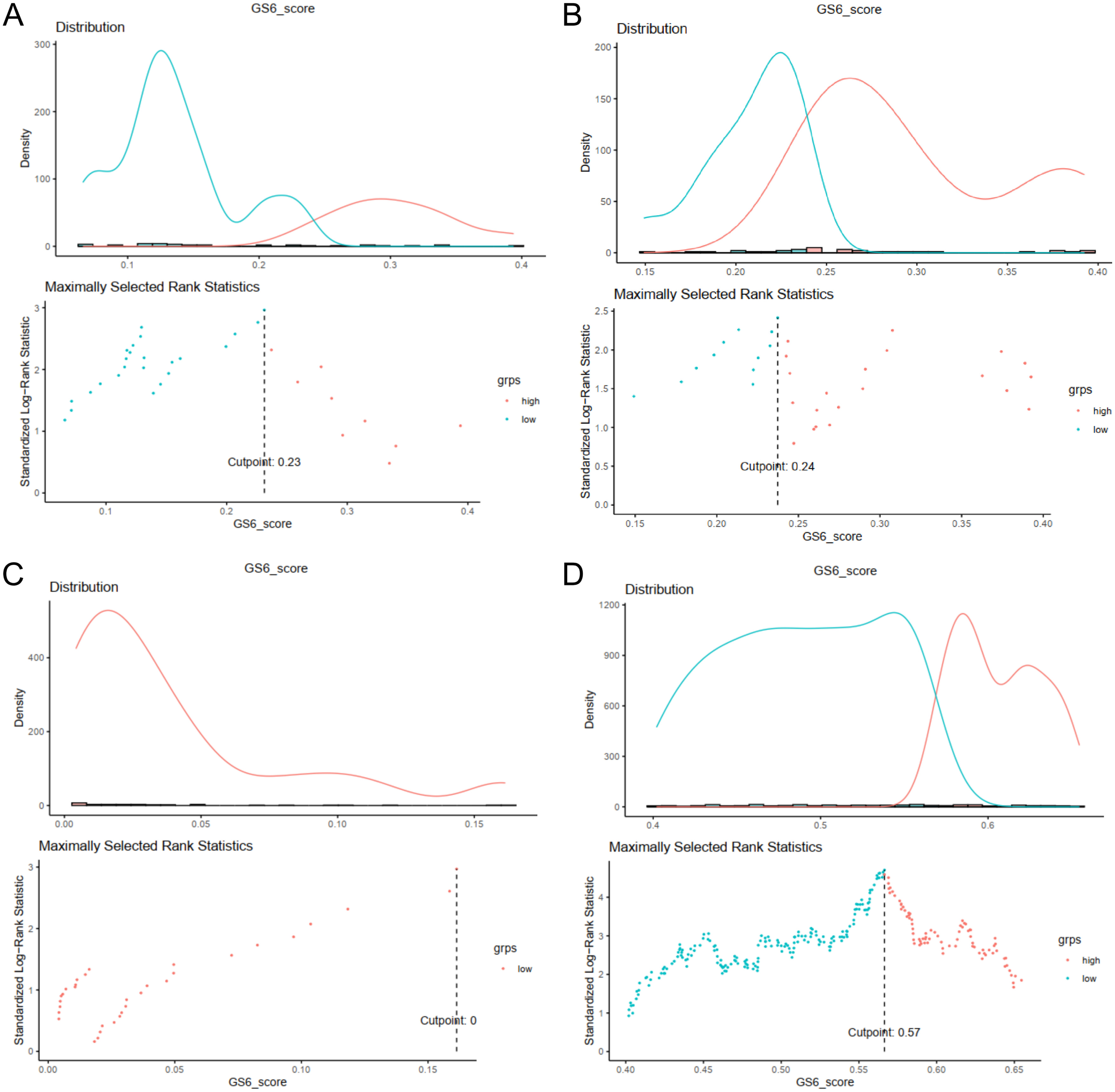

Supplement: Supplementary file 3 [file mmc3.jpg]

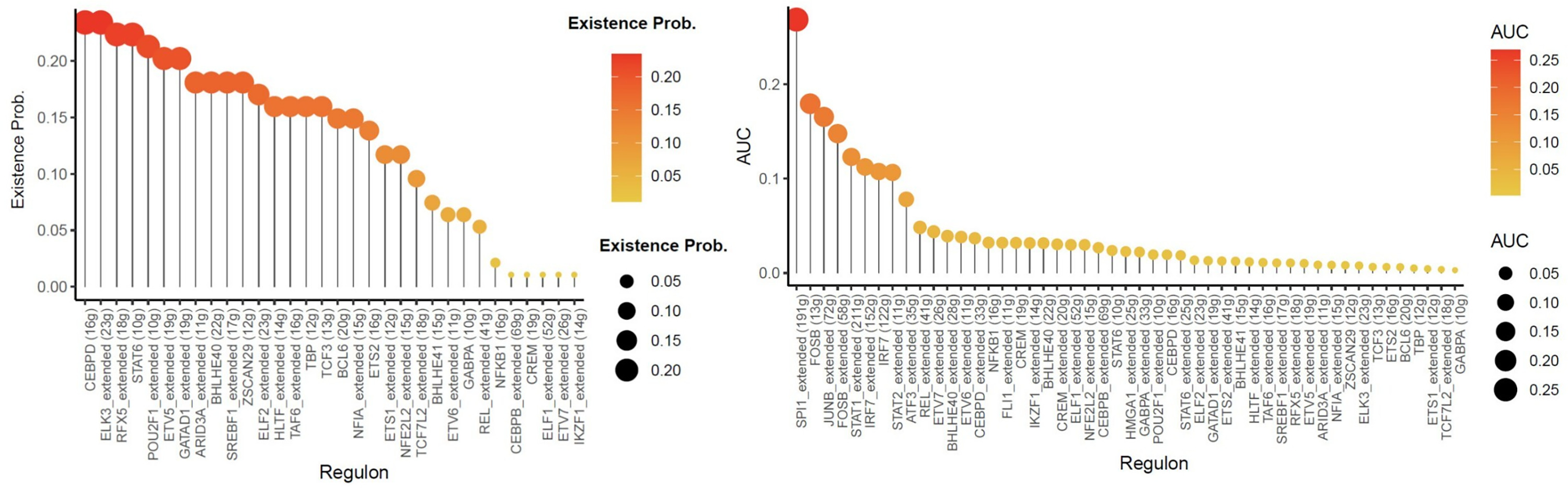

Supplement: Supplementary file 4 [file mmc4.jpg]

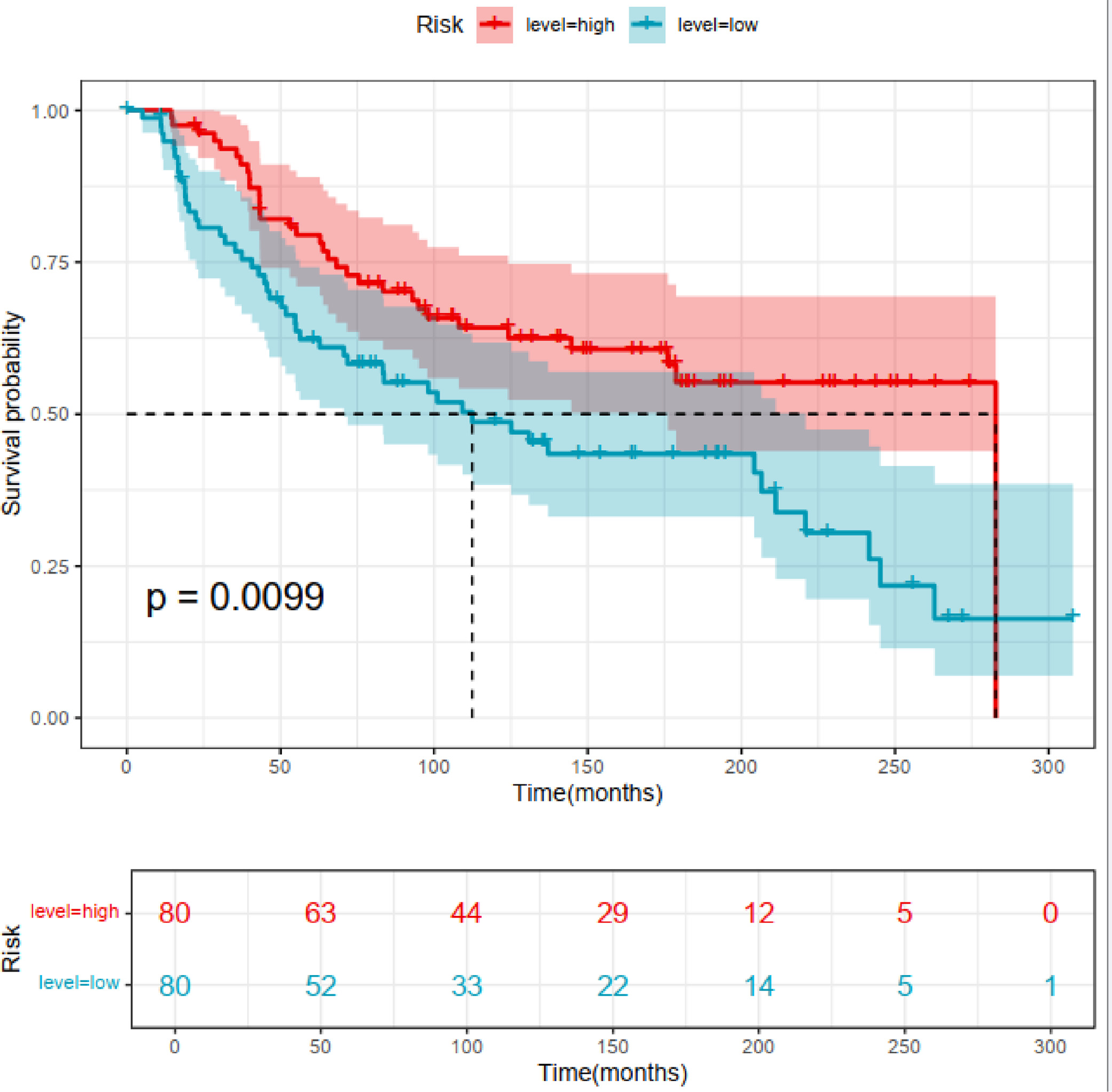

Supplement: Supplementary file 5 [file mmc5.jpg]

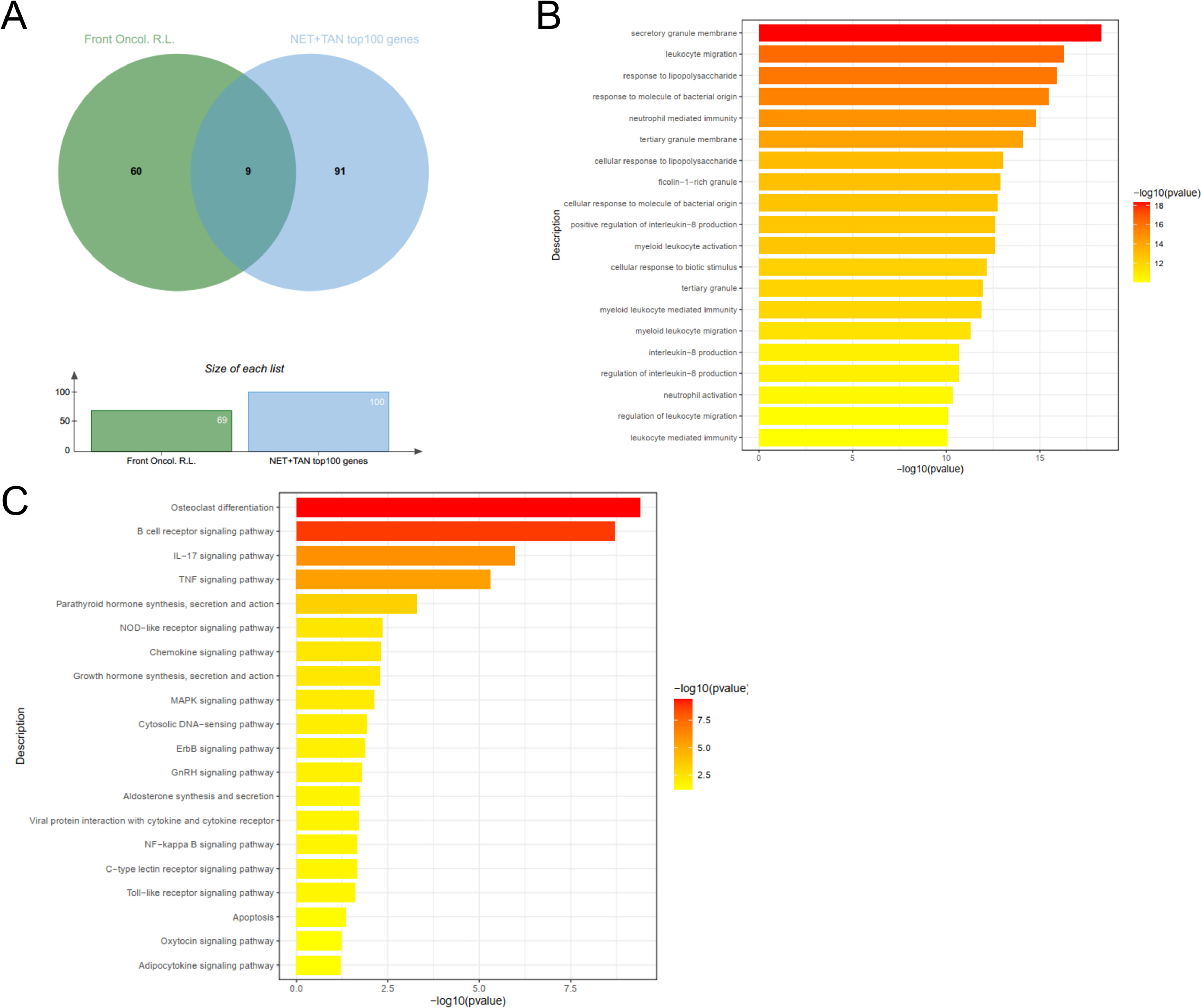

Supplement: Supplementary file 6 [file mmc6.jpg]
