# Supplementary material for: Multi-omics analysis of NET+ TAN explains the immunosuppressive TME and prognosis value of malignant clinical characteristics in TNBC
Source: Transl Oncol. 2026 Feb 10;66:102692. doi: 10.1016/j.tranon.2026.102692 (PMC12914808; doi:10.1016/j.tranon.2026.102692)
Supplement: Supplementary file 7 [file mmc7.docx]

**Supplementary Figures**

Figure S1. Evaluation of Myeloid Cell composition in scRNA-seq and TCGA-BRCA bulkRNA-seq cohort. (A) ssGSEA score of NET+ TAN positive markers from scRNA-seq for each TCGA-TNBC individual was calculated and compared. (B) Cell proportion of myeloid cells in scRNA-seq dataset E-MTAB-8107. The significance mark '*', '**', '***' and '****' represents wilcox p < 0.05, 0.01, 0.001, 0.0001, respectively.

Figure S2. Heatmap visualization ssGSEA score of all cnscerSEA and HALLMARK genesets. Canonical genesets representative of tumor malignancy were highlighted with an asterisk.

Figure S3. Surv_cutpoint function was utilized to divide Overall Survival, Metastasis, Local Recurrence time in mocroarray cohort GSE65194(A-C), and OS in METABRIC cohort.

Figure S4. SCENIC identified TFs in scRNA-seq dataset E-MTAB-8107.

Figure S5. K-M plot for quantile-grouped SLC24A4 expression in METABRIC TNBC patients.

Figure S6. Comparative analysis of NET gene signatures in Li. et al and this study. (A)Venn diagram for NET markers in Li. et al and top 100 positive markers from scRNA-seq data in this study. (B-C) GO enrichment analysis for unique genes from Li et al and this paper.
